# Using Data-Driven Rules to Predict Mortality in Severe Community Acquired Pneumonia

**DOI:** 10.1371/journal.pone.0089053

**Published:** 2014-04-03

**Authors:** Chuang Wu, Roni Rosenfeld, Gilles Clermont

**Affiliations:** 1 School of Computer Science, Carnegie Mellon University, Pittsburgh, Pennsylvania, United States of America; 2 Departments of Critical Care Medicine, Industrial Engineering, and Mathematics, University of Pittsburgh, Pittsburgh, Pennsylvania, United States of America; D'or Institute of Research and Education, Brazil

## Abstract

Prediction of patient-centered outcomes in hospitals is useful for performance benchmarking, resource allocation, and guidance regarding active treatment and withdrawal of care. Yet, their use by clinicians is limited by the complexity of available tools and amount of data required. We propose to use Disjunctive Normal Forms as a novel approach to predict hospital and 90-day mortality from instance-based patient data, comprising demographic, genetic, and physiologic information in a large cohort of patients admitted with severe community acquired pneumonia. We develop two algorithms to efficiently learn Disjunctive Normal Forms, which yield easy-to-interpret rules that explicitly map data to the outcome of interest. Disjunctive Normal Forms achieve higher prediction performance quality compared to a set of state-of-the-art machine learning models, and unveils insights unavailable with standard methods. Disjunctive Normal Forms constitute an intuitive set of prediction rules that could be easily implemented to predict outcomes and guide criteria-based clinical decision making and clinical trial execution, and thus of greater practical usefulness than currently available prediction tools. The Java implementation of the tool JavaDNF will be publicly available.

## Introduction and Background

### Sepsis and Critical Care

Among inflammatory illnesses, pneumonia often presents as sepsis, defined as infection accompanied by systemic signs and symptoms of infection [1], including rapid heart rate, rapid respiratory rate, and fever. Approximately 750,000 patients develop severe sepsis each year in US, with a hospital mortality rate of 28.6%, or 215,000 deaths per year [2]. A significant number of these patients have pneumonia [3]. Interventions for severe sepsis that decrease morbidity and mortality could profoundly impact public health [4]. There is ample pre-clinical and clinical evidence that immunomodulation improves the outcome of patients at higher risks of death, yet pre-clinical data and simulation have also indicated that harm may ensue from targeting some subpopulations of patients [5]–[7]. Early detection of patients at high risk of developing organ dysfunction and death has proved challenging.

Tools to predict the outcomes of critical illness have been developed for three decades [8]–[12]. Most of these prediction tools are logistic regression models, presumably because of their popularity and ease of interpretation of odds ratios associated with predictors of outcome. Yet, logistic regression is intolerant of missing data, does not readily deal with correlated data, and it may be difficult to quickly generate a prediction for the non-expert. A desirable prediction tool should possess the following properties: discrimination (the ability to classify the outcome of patients that who will develop hospital mortality and who will not), learnability (the ability to achieve the discrimination from moderate quantity of data and few features, especially in the early detection of critical care where fewer data are available), completeness (explore the solution space as completely as possible under appropriate assumptions), transparency (not behave as a “black box”), and having the ability to be easily interpretable by the end-user, typically a non-expert.

We propose to use short Disjunctive Normal Form (DNF; “OR” of “AND”) as an appropriate representation of the hypothesis space to predict critical care outcomes because 1) DNF is a high order boolean function that examines potentially complicated relationships between predictors and outcomes, 2) DNF offer great flexibility and allows identification of unforeseen interactions between predictors, 3) DNF is a natural form of knowledge representation for humans to interpret and they provide clinical insights and clear rules to assist in decision making, 4) DNF is scalable to large or small datasets. A short DNF increases interpretability of the rules and mitigates overfitting bias. The aim of this study was to illustrate the ability of DNF to predict hospital and 90-day mortality within 2 days of admission in patients with community acquired pneumonia.

### Related work

Previous models have been limited by retrospective design, [13]–[16] the dependence on large hospitalization data [13]–[19], the lack of interpretability of complex models [16], restricted applicability to single study sites [15], [18], [20], and bias to certain patient populations [15], [16], [18]. Time dependent techniques as alternatives to standard Cox proportional hazard models [21] and dynamic microsimulation [22] have also been published [23] [24]. Both microsimulation and Markov transition kernels derived in these publications are learned from population-level inference and are not instance-based (i.e. patient-specific). We also have the intuition that, outside the framework of a clinical study, clinical data are collected on the basis of perceived clinical need and thus missingness is highly likely not random. Accordingly, there is a very good case to be made that models based on instances might perform better than population models.

From a computational point of view, existing work on outcome prediction models in the clinic learn from a training data set and test their performance using a test data set, such as support vector machine (SVM) regression [25], decision tree classification [26], neural network [27], [28], recursive partitioning [29], linear stepwise regression [30], support vector regression [31], least-squares regression [31] [32], and least angle regression [31]. This body of work focuses on the prediction quality in a cross validation manner. A general flaw associated with models based on these techniques is the absence of clinically meaningful and interpretable functions. Such easily applicable rules would be very desirable indeed in contexts where protocolization of medical decision making, real-time rule-based alerting, or resource allocation is important. Rule induction algorithms, such as decision tree algorithm, [33] and ordered list of classification rules induction [34] can also mine if-then rules. While decision trees can be converted to DNF, the function forms are less flexible due to the constraints of tree structure. Another key difference is that our goal focuses on learning the shortest DNF while decision trees aim at either the fast computational efficiency (heuristic algorithm) or prediction performance (cross-validation test and tree pruning). Sequence analyses using logic regression [35] and Monte Carlo logic regression [36] adaptively identify weighted logic terms that are associated with outcomes; the weakness shared by the random sampling algorithm is the incomplete exploration of the entire hypothesis space.

## Materials and Methods

### The GenIMS study cohort

Patients with community acquired pneumonia (CAP), a common cause of sepsis, were recruited as part of the Genetic and Inflammatory Markers of Sepsis (GenIMS) study, a large, multicenter study of subjects presenting to the EDs of 28 teaching and non-teaching hospitals in 4 regions in the United States (Western Pennsylvania, Connecticut, Michigan, and Tennessee) between November 2001 and November 2003. Eligible subjects were 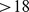 years and had a clinical and radiologic diagnosis of pneumonia, as per the criteria of Fine, et al. [21]. Further details on inclusion and exclusion criteria are provided elsewhere [22]. The GenIMS study was approved by the Institutional Review Boards of the University of Pittsburgh and all participating sites. The current study used fully de-identified data and was approved by the University of Pittsburgh IRB.

Of the 2320 patients enrolled, we restricted our analysis to 1815 subject admitted to the hospital and with measurements of serum inflammatory markers data on enrollment day. Our primary outcomes were all-cause mortality at hospital discharge and at 90 days after enrollment.

### Measurements

The dataset included demographic information, diagnostic information as to bacterial etiology and anatomical site of sepsis, admission APACHE III as an indicator of overall disease severity [23], organ level physiologic variables to quantify organ dysfunction, routine laboratory markers, and interventions. Relevant to our analysis, the inflammatory markers IL-6, IL-10, tumor necrosis factor (TNF), and lipopolysaccharide binding protein (LBP) were collected on days 1, 2, 3, 4, 5, 6, 7, 8, 15, 22 and 30 while patients were still in the intensive care unit. An extended set of coagulation studies was collected on day 1, as well as an array of fluorescent antibody cell sorting (FACS) markers to quantify different immune cell populations on day 1. Finally, DNA information on 27 single nucleotide polymorphism (SNP), each segregating the study population in non-overlapping binary or ternary genotypic categories, was also collected. There were chosen because they were previously shown or suspected to have prognostic value in sepsis [24], [37]–[39].

### Learning the classifier as Disjunctive Normal Form (DNF)

Conceptually DNF is a disjunction of conjunctions where every variable or its negation is represented once in each conjunction. The learning of DNF is a machine learning technique to infer Boolean function relevant with a class of interest. It has been extensively used in electric circuit design, information retrieval [40], chess gaming [41], and so on. Formally, a Disjunctive Normal Form (DNF) is a standardization boolean function, which is a disjunction of conjunctions, where the conjunctions consist of one or more positive and negative literals (statement about the data). Any given boolean function 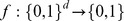 can be converted into an equivalent DNF. The following is an example DNF formula:
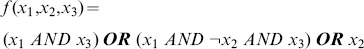
(1)where ‘
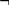
’ denotes negation, and ‘
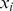
’ denotes a binary literal, indicating whether a particular test “
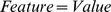
” is true. A DNF formula is essentially a set of Boolean logic if-then rules, describing how the Boolean outcome is calculated based on Boolean inputs.

DNF are traditional binary classifiers that predict Boolean outcomes from instance-based data. The size of DNF functions is 2-dimensional: the number of conjunctive clauses and the maximum number of literals in each clause, thus a DNF is usually represented as k-term n-DNF, where k and n are the number of clauses and maximum number of literals respectively. In DNF learning, k and n are usually regularized because, without constraints, k and n tend to become very large, result in overfitting, thus compromising generalizability. Finding the minimum size DNF formula is a well-known NP-Complete problem [42], [43]. There is no polynomial time learning algorithm, and existing practical solutions usually sacrifice completeness for efficiency. The existing heuristic or approximation approaches fall into deterministic [40], [44], [45] and stochastic algorithms [41], [46]. The deterministic methods include bottom-up schemes (learning clauses first and building DNF in a greedy way) and top-down schemes (converting DNF learning to a Satisfiability problem). Stochastic methods randomly walk through the solution space to search for clauses but are not guaranteed to yield optimal solutions. Our group developed two heuristic algorithms to accelerate the DNF learning by narrowing the solution space under the domain assumptions: standalone DNF learning, and monotone DNF learning (MtDL), described more fully in Appendix S1.

Considered as a core algorithm in concept learning, DNF suffer from shortcomings: 1) the learnability of DNF has been a fundamental and hard problem in computational learning theory for more than two decades, 2) DNF are sensitive to errors in data, as are all Boolean function learning algorithms, 3) without the constraint of size, DNF may suffer from a severe overfitting bias. Our group has been developing algorithms for accelerating and optimizing DNF learning and has been applying the techniques to biomedical data. We specifically focus on short DNF learning to learn meaningful rules as well as to avoid overfitting.

### Model hierarchy and benchmark classifiers

We construct a hierarchy of models 1 to 8 incrementally including features pertaining to different domains of data (Table 1). Model 8 is the most complete model containing all available features; Model 7 is a complete set of features, but restricted to data available only on day 1 of hospital, while Models 1 to 6 include selective domains of features. No data beyond day 2 post-enrollment were included in the predictions.

**Table 1:** Predictors (features) inluded in the different models.

Model	Features included
Model 1	Demographics (age, sex, race, chronic, disease), Macrophysiology (APACHE III score, number of organ system failure on day 1)
Model 2	Demographics, physiology, day 1 cytokines
Model 3	Demographics, physiology, SNP profile
Model 4	Demographics, physiology, day 1 cytokines, SNP profile
Model 5	Demographics, physiology, day 1 cytokines, SNP profile, coagulation data
Model 6	Demographics, physiology, FACS
Model 7	Demographics, physiology, day 1 cytokines, SNP profile, coagulation data, FACS
Model 8	Demographics, physiology, all available cytokines, SNP profile, coagulation data, FACS

To compare the performance of the DNF learning algorithm, a number of other classifiers were constructed. These include simple Logistic Regression, Naive Bayes, SVM, Multi-layer Perceptron (Neural Network), and tree-based algorithms, (e.g. Random Tree, and Random Forest). Prior to classification, all continuous data were discretized in terciles (age), or quartiles (all analytes and APACHE score). For each model, two feature selection algorithms (information gain ranking and chi-square ranking) were run to select a maximum of 15 predictor variables (features). Feature selection was applied using 10-fold cross-validation to mitigate overfitting. Benchmark classifiers used the union of feature sets identified by the selection algorithms.

### Performance metrics

We evaluate the models ability to discriminate outcome by received operating characteristics (ROC) area under the curve. Sensitivity and specificity are also provided. We computed the Brier score as a global measure of calibration. For DNF, we also adapted the Hosmer-Lemeshow H-statistic (AHL) to binary outcomes [47]. Because DNF learning outcomes are either 0s or 1s, we created five bins including a geometrically larger number of predicted deaths. We randomly choose predicted survivors to complete the bins which comprised an approximately equal number of patients. The AHL was then computed as a chi-squared statistic across the five bins [48]. For the probability-based models, e.g., Logistic Regression and SVM, we use their binary outcomes instead of the continuous probability to compute the AHL statistics scores. All metrics are reported in the entire population and in the external validation cohort.

## Results

### Patient characteristics

**Figure 1:**
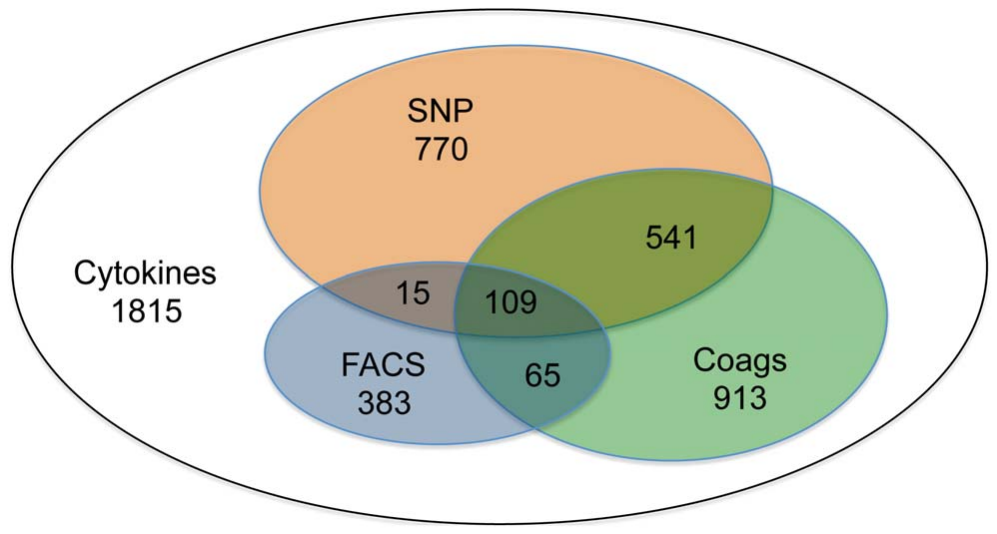
Availability of data across physiologic domains. Of 1815 patients with cytokine data on day 1, much smaller numbers of patients had single nucleotide profiles (SNP), Fluorescent-Antibody Cell Sorting (FACS) measurements of surface markers, or full coagulation studies (Coags)performed.

All 1815 patients had demographic, disease severity and at least two inflammatory markers measured on day 1. The number of patients were different domains of data were available varied and was least for FACS (Figure 1). This distribution strongly determined the hierarchy of models examined. A complete description of cohort demographics and physiology has been published [22].

### Predictors identified by benchmark classifiers

Clinical markers of severity (APACHE score and number of failing organ systems) were the strongest predictors of both hospital and 90-day mortality. Of demographic features, only age and the presence of chronic illness were included in most predictive models. Most SNPs examined were uncorrelated to 90-day mortality, but IL6M174 (GG), L100M1048 (G/T) and MIFM173 (GG) were consistently predictive, even in multivariate models. IL18M137 was less consistently associated with outcome. Features also consistently selected in the hierarchy of models included monocyte positivity for CD-14 and CD-120a, and monocytic and granulocytic positivity for toll-like receptor (TLR)-2. Although it could be that the 10-fold cross-validation procedure admitted significant overfitting (N = 124), it is an interesting hypothesis that the profile of activation of immune cells conveys as much or more information than cytokines and SNP polymorphisms.

### DNF learning algorithm prediction performance

**Figure 2:**
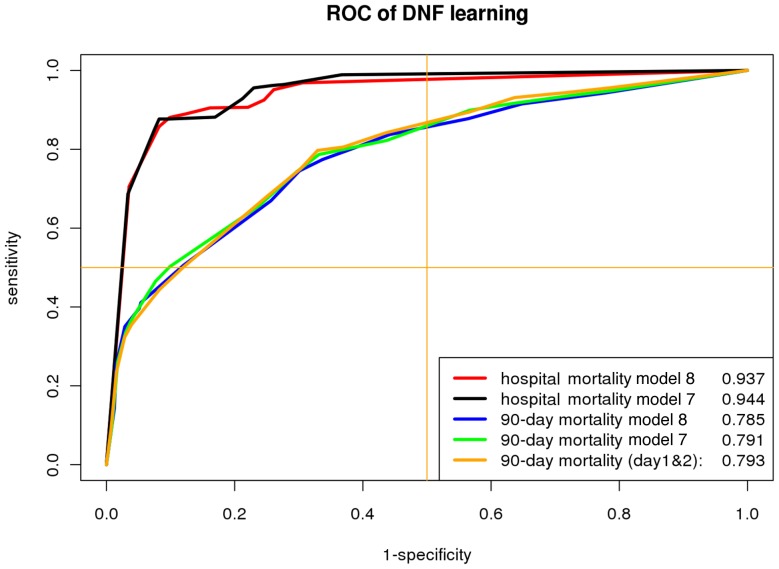
Prediction performance of DNF learning on hospital and 90-day mortality data. 10-fold cross validation is applied to assess the prediction performance of DNF learning on hospital and 90-day mortality, and compare the performance when using the whole feature set (Model 8, see Table 1) and only day 1 (Model 7) and/or day 2 cytokine (Model 7 + day 2 cytokines).

The DNF learning prediction quality is first evaluated by its discrimination. The ROC curve (Figure 2) is generated upon tuning the sensitivity/specificity weights in the optimization objective function. The AUC for hospital mortality dataset in Model 8 is 0.937, which is very similar to the performance obtained with Model 7, suggesting that serum inflammatory markers levels after day 1 do not contribute much to the predictive ability. This is a meaningful result as hospital mortality is by and large determined by data obtained on the first admission day.

90-day mortality is considerably more difficult to predict than hospital mortality with the AUC decreasing to 0.785. We again compare the performance on Model 7, Model 8, and also add day 2 serum inflammatory marker levels to Model 7, without significant improvement in predictive ability (Figure 2). The DNF learning algorithm outperforms other benchmark classifiers built from Model 7 and Model 8 (Table 2), even if Model 8 contains a much more complete set of features; however Naive Bayes and Logistic Regression model prediction performance are lower than that of Model 7 because these two models lack of regularization; Random tree and Random Forests' implementations we used do not implement pruning and result in severe overfitting issues; on the other hand, Boosted Logistic and DNF naturally implements regularizations and perform as well as Model 7 (Table 2).

**Table 2:** Comparative performance of models on predicting 90-day mortality.

Model	NB	SVM	NN	LOG	BL	RT	RF	DNF
Model 1	.740	.716	.746	.748	**.755**	**.705**	**.743**	**.752**
Model 2	.733	.697	.690	.747	**.752**	**.673**	**.681**	**.740**
Model 3	.709	.683	.742	**.762**	**.763**	**.670**	**.696**	**.755**
Model 4	.745	.714	.733	**.762**	**.755**	**.662**	**.711**	**.749**
Model 5	.770	.718	.728	**.774**	**.766**	**.650**	**.654**	**.756**
Model 6	.739	.689	.696	.728	.739	.690	.699	**.759**
Model 7	.783	.747	.751	.785	.766	.701	.715	**.791**
Model 8	.704	.744	.756	.723	.768	.575	.628	**.785**

NB-Naive Bayes, SVM-Support vector machine, NN-neural network, LOG-Logistic regression, BL-Boosted logistic regression, RT-Random tree, RF-Random forest, DNF-Disjunctive Normal Form learning.

When removing features from Model 7 (Models 1 to 6), the DNF learning accuracy decreases (Table 2). DNF learning also outperforms other classifiers on Model 6, suggesting that models which include FACS data perform well despite the modest size of the cohort. For less rich Models 1 to 5, the performances of DNF and benchmark classifiers were comparable, suggesting that richness of the set of features contributes more to the predictive ability of DNF compared to other classifiers. This conjecture could be examined in computational experiments. Interestingly, Logistic Regression-based classifiers performed consistently better than other benchmark classifiers through Model 5 (Table 2).

### DNF learning algorithm external validation

To evaluate the external validity of predictions from DNF learning, we developed models using patients from a random subset of 27 hospitals, comprising approximately two-thirds of the patients. The prediction performance of DNF rules are then tested on patients from the remaining six hospitals, where the numbers of patients per hospital varied between 1 to 343.

Using 90-mortality as the outcome of interest the DNF learning ROC achieves 0.789 which is similar to that we learned in cross-validation over the entire cohort when using all the features. The external validation performance of DNF learning compared advantageously with that of benchmark models (Table 3). Of note, DNF learning was the best calibrated model (AHL = 9.06, p = 0.06 with 4 df).

**Table 3:** Comparative performance of models on predicting 90-day mortality.

Scores	NB	SVM	NN	LOG	BL	RT	RF	DNF
ROC	.747	.752	.757	.738	.748	.655	.698	**.789**
1 - Brier Score	.712	.750	.844	.822	.867	.792	.874	**.891**

NB-Naive Bayes, SVM-Support vector machine, NN-neural network, LOG-Logistic regression, BL-Boosted logistic regression, RT-Random tree, RF-Random forest, DNF-Disjunctive Normal Form learning.

### Specific rules learned from the data

The DNF learning algorithm simultaneously optimizes the prediction quality and minimizes the length of DNF functions, because without constraining the function length, the DNF functions can be complicated and lead to severe over-fitting problems. The DNF learning algorithms aim to learn the shortest functions (see section 0 for the definition of the function length), i.e. the most generic functions extracted from the data that can discriminate the mortality outcomes. The DNF learned to predict hospital mortality is:
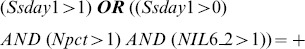
(2)


Where 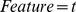 means the value of the feature falls into group *t*; 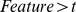 means the feature value is larger than that of group t. Recall that the feature values are discretized into 3 to 5 groups, and the group values are indexed from 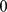 to 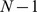 where N is the number of groups. The full explanation of literals appeared in this study is shown in Table 4.

**Table 4:** DNF literals explanation.

literal	meaning	value type	num of value groups
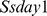	Presence of some organ dysfunction on day 1 [49]	integer	5*
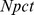	Quartile of procalcitonin [50]	integer	5
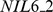	Quartile of the inflammatory marker IL-6 on the second day of admission	integer	5
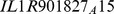	Genetic polymorphism of IL-1 receptor antagonist protein	Gene	3
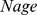	Quartile of age	integer	5
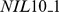	Quartile of the inflammatory marker IL-10 on the day of admission	integer	5
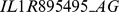	Genetic polymorphism of IL-1 receptor antagonist protein	Gene	5
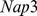	Quartile of APACHE III score	integer	5
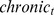	Burden of chronic illness, as determined by the Charlson index [51]	integer	5
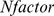	Quartile of coagulation Factor IX activity	integer	5

Note.

*: when missing values present in the data, they are treated as a literal, but they are never selected in the DNF learning.

Function (1) indicates that if either one of two conditions is satisfied, the outcome is predicted to be hospital death, where the two conditions are 1) 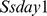 value is larger than 1 (failure in more than one organ system), or 2) 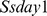 value is larger than 0 AND 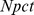 value is larger than 1 AND 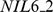 value (quartile of IL-6 levels on the second day) is larger than 1. The positive symbol on the right side of function (1) is positive label, i.e., hospital mortality. Since all the DNF predict positive class, the ‘+’ symbol on the right side is replaced with the sensitivity/specificity metrics of the DNF. For representation purposes a DNF will be written as DNF = sensitivity/specificity, and the above function is now:
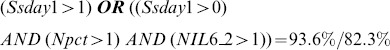
(3)


This DNF contains 2 terms of 4 literals covering 3 different features: 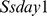, 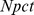, and 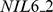, comprising only 3% of all features available in the data, suggesting that DNF functions discriminate the outcomes by only using a small fraction of the feature sets (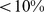 features in all cases).

**Figure 3:**
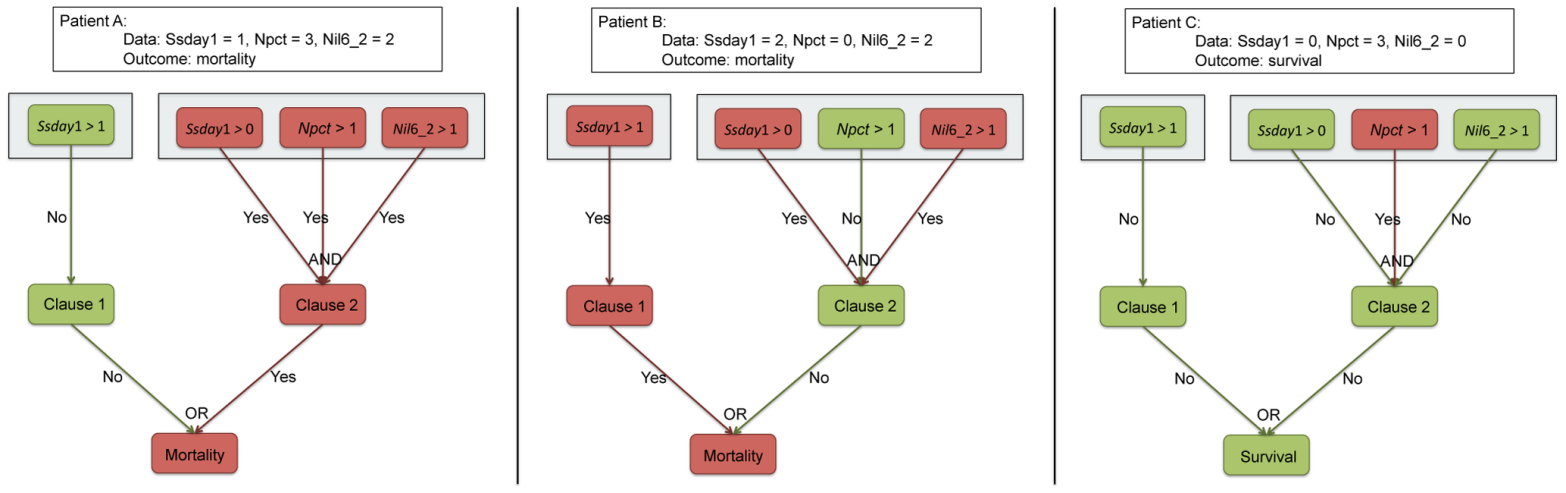
Interpreting DNF models on three patients. The prediction procedure of DNF is represented in three layers: the top layer is the DNF itself; the middle layer is the clause level; and the bottom layer is the final outcome. Red color rectangles indicate that patient data is above the threshold and a severity condition is met; green rectangles indicate that patient data is below and the condition is not met. Three example patients are shown. For patient A, 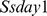, 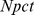 and 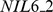 are all above the threshold and results in a positive Clause 2 so the predicted outcome is mortality. For patient B, Clause 2 is negative due to the low 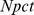 (procalcitonin in the lowest quartile); however high 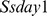 turns on Clause 1 and predicts mortality too. Patient C has high 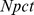 but it is not sufficient to turn on either Clause 1 or 2 and she is therefore predicted to survive.

The prediction procedure implied by a DNF (3) is illustrated in Figure 3. The prediction procedure of DNF is represented in three layers: the top layer is the DNF itself; the middle layer is the clause level; and the bottom layer is the final outcome. Red color rectangles indicate that patient data is above the threshold and a severity condition is met; green rectangles indicate that patient data is below and the condition is not met. Three example patients are shown. For patient A, 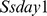, 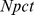 and 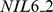 are all above the threshold and results in a positive Clause 2 so the predicted outcome is mortality. For patient B, Clause 2 is negative due to the low 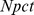 (procalcitonin in the lowest quartile); however high 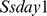 turns on Clause 1 and predicts mortality too. Patient C has high 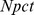 but it is not sufficient to turn on either Clause 1 or 2 and she is therefore predicted to survive.

The DNF learned from the data are shown in Table 5. For hospital mortality, 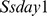 is a strong predictor. A high level of 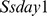 is associated with high risk of mortality. 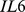 and 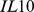 are strong predictors too, and appear to be consistently predictive, which can possibly support the concept that total inflammation, as opposed to a balance between pro-inflammation and anti-inflammation, is predictive of outcome [21]. 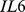 on day 2 turns out to be a strong predictor, yet needs two other conditions to also be present (Equation (1) in Table 5). In Model 7, 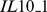 is selected instead, and it needs 3 other conditions too: 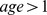, 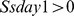 and 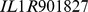 SNP is not A/G (Equation (2) in Table 5).

**Table 5:** DNF of the patient mortality.

Hospmort mortality (model 8)	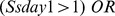 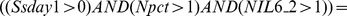 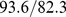
Hospmort mortality (model 7)	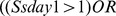 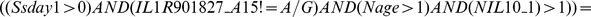 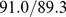
90-day mortality (model 8)	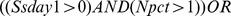 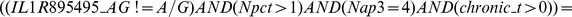 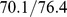
90-day mortality (model 7)	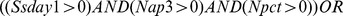 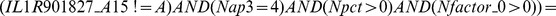 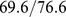

To predict 90-day mortality, the number of terms in DNF increases to 5, and the sensitivity decreases to 80%, suggesting that 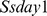 is not as strong a predictor of 90-day mortality as it is of hospital mortality. In Model 8, 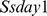 combines with 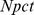 factor to form a single clause, and in Model 7 it needs 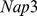. Higher 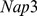 is also an indication of high death risk. Interestingly although SNP generally has low correlation with the 90-day mortality, 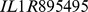 and 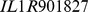 are learned in the DNF.

The highest discriminator of poor outcome was the day 1 to day 2 trend in the product of IL-10 and IL-6. Trends in day 1 to day 2 TNF, IL-10, IL-6, were also retained in the models. This is a very interesting, and somewhat refreshing observation, raising the hypothesis that interventions significantly impacting early cytokine profiles might indicate biological activity resulting in more favorable long-term outcome.

In the external validation, the DNF learned from the development set is:
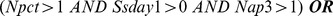


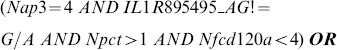


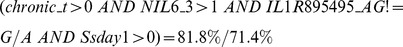
(4)


The first two clauses are similar to those learned in Table 5, which indicated that 1) the process of DNF learning is robust in identifying predictive rules if data used in development is consistent with population data, and that 2) correlations in data may allow similar, but not identical rules, when different development sets are selected.

## Discussion and Conclusion

We present a new class of models, DNF learning, which produce data-driven rules predicting mortality in patients hospitalized with severe community acquired pneumonia (see Appendix S1 for details). A distinctive feature of DNF, compared to commonly presented prediction models, is that the resulting rules are readily interpreted by clinicians and can be used to enhance clinical decision making in a variety of contexts. These rules are created under the assumption that DNF are an appropriate representation of the manner data relate to outcome in severe community acquired phenomena. In other words, several alternative (disjunctions) mechanisms can contribute to the outcome, each mechanisms represented by the conjunction of conditions. The assumption is clinically plausible and important as we develop algorithms to compute the DNF, because it reduces the hypothesis space greatly and makes the computational hard problem solvable in reasonable time. We demonstrated learning efficiency and consistency on simulated sequences, showed the strength of the methods in learning meaningful mapping functions and showed superior prediction accuracy compared to other machine learning methods on real clinical data.

The use of DNF as a prediction tool has several strengths. Prediction rules are intuitive and easy to apply at the bedside (Figure 3). They could be easily interfaced with the electronic health record. Because a rule is comprised of separate disjunctive statements, each or which can be true or false, its veracity can typically be assessed even if partial data is available, and very soon following an initial assessment of the patient. A popular mortality prediction model, APACHE [49], requires 24 hours of observation before formulating a prediction. Another popular tool, MPM [50], uses information available upon initial encounter, but is less accurate and requires many more data elements to formulate a prediction. Prediction models not based on logistic regression are essentially black-box classifiers which provide little insight as to which feature drives the prediction. In this regard, DNF are very transparent in their use of data to generate a prediction.

We aimed to learn the minimum size DNF in spite of the fact that the exact learning task is NP-complete [42], [43]. Compared to existing heuristic algorithms that only focus on learning time and learnability [40], [41], [44]–[46], we exploit domain knowledge and develop efficient exhaustive algorithms to learn the shortest DNF. We also applied a number of techniques to accelerate the DNF learning process (see Appendix S1 for details), including setting the maximum length of clauses in standalone algorithm, using feature selector (CF) in MtDL to narrow down the searching space, equivalence filtering of the clauses, and extending both algorithms to greedy versions. This enables the algorithms to run efficiently on large datasets. The DNF learning algorithms are also powerful in extracting DNF from only a small numbers of sequences where the data are reliable.

The approach achieves equivalent or higher prediction performance compared to a set of state-of-the-art machine learning models, and unveils insights unavailable with standard methods. For example, we have shown that although predictive on their own, the added benefit of genetic and cytokine data over physiology and demographics-based classifiers was not spectacular in identifying poor long-term outcome. It also appears that, if one were to choose between a serum assay and a DNA profile (or SNP screen) as an early predictor of outcome, both convey comparable information with the possible exception of the product of serum levels of 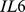 and 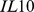, plausibly a (quite naive) integrator of the magnitude of the inflammatory response. There are no currently available point-of-care kits to measure cytokine panels reliably, although a rapid kit exists for IL-6. The same is true of SNP profiling. Our exploration suggests that we probably do not need both a cytokine and SNP profile at this time, but the jury is certainly not out. Yet, it cannot be anticipated that such detailed physiotyping will be commonly performed at the bedside in the foreseeable future. Therefore, it would seem appropriate to expand data available to the DNF algorithms to include a larger overlap with data used by currently available mortality prediction tools. Indeed, one could conceive of DNF rules as representing phenotypes, confined to data that is already available, and that could be refined if more data were available to develop a more complete set of rules. The level of sophistication with which these phenotypes would be described would increase from purely clinical, to phenotypes characterized by a combination of clinical, laboratory, and genetic markers.

Our exploration was limited to 27 SNPs and 3 cytokines, and several leukocytic surface markers in a subset of the population therefore our representation of the cellular and genetic component to physiotyping is very limited. Other analytes are now becoming available in this database, including SNPs for coagulation genes, which are definitely strong predictors of outcome. This can be understood mechanistically when that considering excessive activation of coagulation, with subsequent microthrombosis and perfusion deficit, is a plausible cause of cellular energetic failure with ensuing organ dysfunction [11].

It can be argued that 90-day mortality is an inappropriate outcome and that one would expect early physiotyping to perform better on predicting outcome on a shorter time scale. However, it is apparent, especially in this dataset that our current concept of what constitute acute illness extends well beyond the intensive care unit, or a specific hospitalization episode [51], [52]. It makes entire sense that wider genetic screens might be more predictive than early physiology in teasing late death. Different classes of predicative models are required to tease out time-varying hazard ratios [53]. Such a study would be a natural extension of this work. It could also be argued that predicting mortality does not mean the ability to predict response to treatment, a holy grail of acute care medicine. Any signal in the possible effectiveness of immunomodulatory therapies has been observed in the sickest individuals. [54], [55], suggesting the relevance of more detailed physiotyping in the prediction of the response to treatment. This is also suggested by in silico studies [7]. The DNF formulation can generally applied to a variety of outcomes of clinical interest. For example, enrollment and decision points in clinical trials are often criteria-based. The applications of data-driven rules computed from DNF learning to the profiles of patients currently screened or enrolled in clinical trials could be quite helpful to assist clinical trial design, enrich enrollment, or eventually adapt design based on observed response.

In conclusion, we presented DNF as a novel prediction tool which perform comparably or better than currently available tools to predict outcome in patients with hospitalized community acquired pneumonia, and which presents the added advantage to be criteria-based and easily implemented as a decision support system at the bedside. We believe DNF are generally applicable to a range of clinically relevant patient-centered outcomes. Despite its apparent simplicity, DNF do require the input of expert quantitative scientists to develop and implement.

## Supporting Information

Appendix S1 (PDF)

Table S1 **Clause learning algorithm.**
(PDF)

Table S2 **DNF learning algorithm.**
(PDF)

Table S3 **Monotone DNF learning algorithm.**
(PDF)
