# Supplementary material for: Using Data-Driven Rules to Predict Mortality in Severe Community Acquired Pneumonia
Source: PLoS One. 2014 Apr 3;9(4):e89053. doi: 10.1371/journal.pone.0089053 (PMC3974677; doi:10.1371/journal.pone.0089053)
Supplement: Appendix S1 — (PDF) [file pone.0089053.s001.pdf]

Appendix

Standalone DNF learning

Valiant [S1] showed that for every constant $k \geq 1$, k term DNF can be PAC learned in polynomial time by k-CNF (Conjunctive Normal Form), i.e. CNFs with at most k literals in each clause. k-term DNF learning is essentially a combinatorial problem. The standalone DNF learning algorithm first deterministically learns a set of conjunctive clauses with maximum length of k (Table S1), and then converts the DNF learning process into the typical SET-COVER problem (Table S2): finding the minimum number of sets that cover all the positively labeled data points. The SET-COVER problem is again NP-Complete, by limiting the maximum clause length, for typical problem settings the number of clauses is usually manageable and the SET-COVER can be exhaustively completed. Typically, the number of possible clauses of size k is up to L x k, where L is the number of features. Depending on the diversity of the data, the actual number of clauses that appear in the dataset is much smaller. After equivalence filtering, the number of learned clauses is usually on the order of several hundreds in the clinical domain. The standalone algorithm can efficiently infer DNF from small (a few sequences) to medium size (hundreds of sequences) datasets, or large conserved datasets.

Monotone DNF learning after feature selection (MtDL)

MtDL utilizes feature selection to narrow the feature space and infer DNF within the space. The choice of feature selector is critical to the MtDL algorithm. The best feature selector needs to guarantee that the selected feature space is a superset of the DNF solution space, and best limit the feature space for efficient learning. The Combinatorial Filtering (CF) algorithm we developed [S2] works seamlessly with MtDL as a feature selector. CF() efficiently identifies the smallest set of features that completely explains the differences between classes, such that MtDL is guaranteed to learn DNF. In this study, MtDL always runs together with CF() to infer DNF (Table S3). Notwithstanding, other feature selection methods, such as LASSO, Logistic Regression with regularization, or dimension reduction methods like PCA, are also good candidate selectors, but in these cases, the coverage threshold might need to be set (see section Avoiding over-fitting and robustness to noise).

Upon completion of feature selection, the MtDL enumerates literals in the selected features and then combines them into conjunctive clauses (Table S3). MtDL differs from the standalone algorithm in that it does not limit the maximum size of clauses but completely considers all 2^M possible combinations. Take a typical example: once L features are selected in step 1, there should be at most $M = V$ x L possible literals, where V is the size of value space. Because literals from the same feature will not appear in the same conjunctive clause, we do not need to consider all 2^M combinations, but only combinatorially choose up to L literals from M. Hence the total number of clauses is at most $N = (MchooseL)$. In reality, depending on the divergence and the amount of the data, the actually number of possible literals is always much smaller. Furthermore, in step 4, the N clauses will be pre-filtered by removing clauses that cover any negative sequences. When the clause pool is ready, in step 5 the algorithm incrementally constructs the combination of clauses to be candidate DNF and examines the coverage of data points. MtDL starts from one clause, and checks the next larger number if no solution is found. The algorithm terminates when the DNF exclusively cover all positively labeled data points but not any of the negatively labeled ones.

Greedy versions of both algorithms

The greedy versions of the two DNF learning algorithms are designed to rapidly learn DNF from very large datasets. In the DNF construction step, instead of exhaustively combining all clauses to construct

DNF, the greedy algorithms iteratively select the clause that covers the largest number of the uncovered positive sequences and zero negative sequences until all positive sequences are covered.

Equivalence filtering

Computationally equivalent clauses cover the same set of sequences while differing in their composition literals. Replacing one clause with its equivalent clauses in a DNF will not change the predictions of the DNF on the same training set. Equivalent clauses are very common in clinical datasets; therefore, during DNF learning process equivalent clauses are filtered and only one of them is used as the representative to construct DNF. By using equivalence filtering the DNF learning running time is greatly reduced. Note that the equivalence filtering is only for computational efficiency purpose. After learning DNF, all clauses that have equivalent clauses will be expanded to recover all the DNF.

Avoiding over-fitting and robustness to noise

We used pruning and threshold setting to mitigate over-fitting and make the algorithms improving robustness.

1. **Pruning clauses**: similar to the pruning of decision tree, clauses that only cover a small number of sequences may be pruned if removing them results in an increase of the prediction accuracy on the test dataset. The advantages of pruning are:

   - avoiding over-fitting, because irrelevant clauses are removed,
   - shortening DNF, which makes them easier to understand and more biologically meaningful and
   - increasing robustness to noise, because pruned DNF ignore the clauses/literals rendered meaningless by noise.

2. **Threshold setting**: setting thresholds of the fractions of the sequences that the learned DNF(s) cover: the DNF learning algorithms can be easily modified to terminate when at least a fraction p of the positive sequences are covered, and at most a fraction n of the negative sequences can be covered by the DNF. The thresholds p and n are determined by cross-validation. Similar to pruning, threshold setting can also avoid over-fitting, learn shorter DNF and be robust to noise. One advantage of threshold setting over pruning is that the former technique usually achieves better prediction quality.

Extension to multiple class data

The algorithm is applicable to multiple class data by running the algorithm multiple times. Each time, one of the classes is designated as the positive class and the rest are merged as negative class.

DNF learning algorithm consistency and efficiency

The DNF learning algorithms are first validated on simulated clinical data to assess the consistency and learning efficiency in a practical way. When generating the simulated data a new hypothetical DNF is proposed in each study, e.g. $((age > 30\ AND\ Sepsis > 1)\ \boldsymbol{OR}\ NIL6\_2 > 1 = mortality)$, see Table 4 for the explanation of DNF functions) and the data is randomly produced following the features distributions in the real patient data and labeled according to the target function. When learning the DNF the positively labeled and negatively labeled data are shuffled and used in an incremental way to monitor the solution space size against the number of data used. The step is repeated 20 times and

the DNF learning algorithm converge to a single DNF solution with 50 positively labeled data and 50 negatively labeled data. Importantly the learned single DNF is consistent with the target function in all of the repeats.

Software and guideline

The Java software is publicly available on github: https://github.com/chuangwoo/JavaDNF

- Support platforms: Linux, Windows, Mac
- Requirements: Java >= 1.6, maven.

To install:

```
$ git clone https://github.com/chuangwoo/JavaDNF
$ mvn project
```

Running scripts are also available in the git pool.

References

S1 Valiant L. G. (1984) A theory of the learnable. Proceedings of the sixteenth annual ACM symposium on Theory of computing table of contents. 436-445.

S2 Wu C, Walsh A, Rosenfeld R. (2010) Identification of Viral Protein Genotypic Determinants using Combinatorial Filtering and Active Learning. IEEE-BIBE 162-167.
