# Supplementary material for: Using Data-Driven Rules to Predict Mortality in Severe Community Acquired Pneumonia
Source: PLoS One. 2014 Apr 3;9(4):e89053. doi: 10.1371/journal.pone.0089053 (PMC3974677; doi:10.1371/journal.pone.0089053)
Supplement: Table S1 — Clause learning algorithm. (PDF) [file pone.0089053.s002.pdf]

Table S1. Clause learning algorithm

Clause Learning Algorithm (S, k):
Input:

A set **S** of already-available labeled sequences
k: assumed upper-bound length of clauses (a small positive integer)

Steps:

1. Enumerate all combination of literals to form conjunction clauses
2. Record the set of (positive and negative) sequences that each clause covers (n_j^+, n_j^-)

Output:

The set of clauses C and the corresponding sequence index sets (N^+, N^-)
