# Supplementary material for: Using Data-Driven Rules to Predict Mortality in Severe Community Acquired Pneumonia
Source: PLoS One. 2014 Apr 3;9(4):e89053. doi: 10.1371/journal.pone.0089053 (PMC3974677; doi:10.1371/journal.pone.0089053)
Supplement: Table S2 — DNF learning algorithm. (PDF) [file pone.0089053.s003.pdf]

Table S2. DNF learning algorithm

Disjunctive Normal Form Learning Algorithm(C, n^+):
Input:

A set **C** of clauses
n^+: the set of positive sequence index to be covered by the clauses

Steps:

1. Euivalence filtering
2. Among the clauses that cover only the positive sequences, find a minimum set of clauses that cover all the positive sequences:
   2a. start from the clauses that cover the positive sequences which are rarely covered by other clauses
   2b. repeat 2a recursively until all the positive sequences n^+ are covered

Output:

The set of the shortest DNF
