# Supplementary material for: Using Data-Driven Rules to Predict Mortality in Severe Community Acquired Pneumonia
Source: PLoS One. 2014 Apr 3;9(4):e89053. doi: 10.1371/journal.pone.0089053 (PMC3974677; doi:10.1371/journal.pone.0089053)
Supplement: Table S3 — Monotone DNF learning algorithm. (PDF) [file pone.0089053.s004.pdf]

Table S3. Monotone DNF learning algorithm

Monotone DNF Learner (*F*, *S*):

Input:

F: A set of selected features (by CF(), for example)

S: the labeled training datasets

Steps:

1. Construct {L}, the list of literals in the features (e.g. 5A).
2. Throw out L that does not cover any positive sequences.
3. Combinatorial construct {Clauses}, the list of conjunctive clauses from {L}, (e.g. $5A\ AND\ 8C$). The possible combinations are $|L|$ chooses $1, 2, .., |F|$.
4. Throw out the conjunctive clauses that cover any negative sequences.
5. Incrementally construct {DNF}, the list of disjunctive normal form that covers all positive sequences but no negative sequences: starts from 1 clause, construct DNF from {Clauses}, try the next larger number if no solution learned.

Output:

The set of the shortest DNF
